# Complete genome sequence analysis of the multidrug resistant *Aeromonas veronii* isolated for the first time from stinging catfish (Shing fish) in Bangladesh

**DOI:** 10.5455/javar.2023.j711

**Published:** 2023-09-30

**Authors:** Mohummad Muklesur Rahman, Mohammad Sadekuzzaman, Md. Asikur Rahman, Mahbubul Pratik Siddique, Mohammad Asir Uddin, Md. Enamul Haque, Md. Golam Azam Chowdhury, A. K. M. Khasruzzaman, Md. Tanvir Rahman, Muhammad Tofazzal Hossain, Md. Alimul Islam

**Affiliations:** 1 Department of Microbiology and Hygiene, Bangladesh Agricultural University, Mymensingh, Bangladesh; 2 Central Disease Investigation Laboratory, Department of Livestock Services, Dhaka, Bangladesh; 3 Bangladesh Fisheries Research Institute, Mymensingh, Bangladesh

**Keywords:** *Aeromonas veronii*, antimicrobial resistance, fish, phylogeny, whole genome

## Abstract

**Objective:**

Whole genome sequencing (WGS) of *Aeromonas veronii* Alim_AV_1000 isolated from ulcerative lesions of Shing fish (stringing catfish; *Heteropneustes fossilis*) was performed during the outbreak year 2021.

**Materials and Methods:**

Using next-generation sequencing (Illumina) technology, WGS was accomplished, resulting in the sequencing, assembly, and analysis of the entire genome of the *A. veronii* strain. Moreover, the genomic features, virulence factors, antimicrobial resistome, and phylogenetic analysis for the molecular evolution of this strain were also examined.

**Results:**

The genome size of the *A. veronii* Alim_AV_1000 strain was 4,494,515 bp, with an average G+C content of 58.87%. Annotation revealed the known transporters and genes linked to virulence, drug targets, and antimicrobial resistance.

**Conclusion:**

The findings of the phylogenetic analysis revealed that the strain of the present study has a close relationship with the China strain TH0426 and strain B56. This study provides novel information on *A. veronii* isolated from Shing fish in Bangladesh.

## Introduction

Bangladesh is currently ranked third in the world for inland fish output and fifth for production from aquaculture. Fish production in Bangladesh is presently at a level where it meets all of the country’s demands, and the country is beginning to gain recognition on a global scale as one of the countries that produces the most fish overall [1]. Due to the increasing demand from customers, intensive fish farming methods are becoming increasingly prevalent in aquaculture [2]. On the other hand, this approach may result in imbalanced physiological circumstances and entail the potential for infectious disease outbreaks, ultimately becoming a potential threat to the entire aquaculture industry [3].

Among bacterial pathogens, *Aeromonas veronii* [*A. veronii*, responsible bacterial agent for motile Aeromonas septicemia (MAS)], a Gram-negative, rod-shaped, motile bacterial pathogen, is considered the leading one [4]. In addition to aquaculture, this infection has been shown to cause infections in humans (namely gastroenteritis), amphibians, and other aquatic creatures [4]. Skin ulcers, fin rot, tail rot, stomach distension, exophthalmia, and hemorrhagic septicemia are all symptoms of an *A. veronii *infection in fish, with a mortality rate of 10%–100% [4,5]. The clinical signs are not homogenous across infected fish [4], and disease outbreaks have been reported from China in cultured channel catfish [6] and from Saudi Arabia in tilapia [7].

Several techniques for strain characterization have been used, including serotyping, antimicrobial susceptibility testing, and mass spectrometry-based methods, considered the gold standard for bacterial phenotypic characterization [8]. Recently, whole genome sequencing (WGS) has been considered the fastest-evolving, most familiar, and widely used technique to determine the genetic makeup of organisms [9]. The high discriminative power of WGS also enables the determination of phylogeny and the identification of subspecies. It is, hence, widely used to study strains of origin in all aspects, *viz*., humans, animals, and the environment, including foods [10].

*Aeromonas veronii* strains collected from various sources, such as fish, dairy cattle, humans, and the environment, have been previously sequenced [11–13]. However, in Bangladesh, limited genomic information is available on what types of *A. veronii* strains are circulating. Therefore, for a better understanding of the *A. veronii* pathogenic strain in fish, there is a need to explore genomic analysis in the context of Bangladesh. In this study, the whole genome of the *A. veronii* strain of a stinging catfish (Shing fish; *Heteropneustes fossilis*) was sequenced. The genomic characteristics, virulence factors, resistome profile, and phylogenetic relationships with other reported strains were also analyzed.

## Materials and Methods

### Isolation and identification of A. veronii strain

*Aeromonas veronii *strain was isolated from the liver of a stinging catfish in the Mymensingh district of Bangladesh. The liver samples were collected aseptically from infected fish. Bacterial isolation was performed on Tryptic Soy agar medium through streaking of overnight cultured alkaline peptone broth, and then incubation was done (temperature: 37ºC; duration: 24 h). Molecular identification of the bacterium, which revealed characteristic colonies, was done through matrix-assisted laser desorption ionization-time of flight mass spectrometry (MALDI-TOF MS) (Bruker Daltonics, Bremen, Germany), commercially available from the Quality Control Laboratory, Department of Livestock Services, Savar, Dhaka, Bangladesh.

### Genomic extraction

The pure colony was further cultured in TS broth (Oxoid, UK) at 37ºC shaking at 150 rpm. The Wizard^®^ Genomic DNA Purification Kit (Promega Corporation, 2800 Woods Hollow Road, Madison, WI 53711-5399) was utilized following the manufacturer’s instructions to extract bacterial genomic DNA.

### Genome sequencing, assembly, and analysis

The Illumina DNA Prep Library Preparation Kit (Illumina, Inc., 5200 Illumina Way, San Diego, CA) was used to generate sequencing libraries according to the manufacturer’s recommendations and sequenced them on a Novaseq sequencer using single-end and paired-end sequencing. Following adapter trimming and quality filtering, the reads were examined through FastQC and assembled using MEGAHIT software. Genome sequences were analyzed using the Illumina Base Space Sequence Hub software and submitted to the Bacterial and Viral Bioinformatics Resource Center pipeline for comprehensive genome analysis. Genome synteny between this *A. veronii* isolate and a reference genome was conducted using Mauve v 2.3.1 [14]. For all Softwares, the default parameters were used unless otherwise stated. Putative virulence factors were identified using an integrated and comprehensive online resource named VFanalyzer and the virulence factor database (VFDB) [15].

### Multilocus sequence typing

The sequence query tool was employed to determine the Alim_AV_1000 genome’s sequence type (ST) by comparing it to the pubMLST database, available on the pubMLST website (https://pubmlst.org/aeromonas/).

### Proteome analysis

Homologous protein sequences between *A. veronii* strain Alim_AV_1000 and the reference strain (NZ_CP044060.1) were identified using the PARTIC proteome comparison tool [16].

### Prophage determination

The PHAge Search Tool Enhanced Release (PHASTER) search tool was employed to detect potential prophages [17]. For this purpose, the nucleotide sequences of the *A. veronii* genome were served as an input file to the PHASTER server. The computed and analyzed results were described under three categories based on the scores obtained: a score >90 indicates an entire phage; a score <70–90> is considered dubious; and a score <70 suggests an incomplete phage element.

### Determination of the antibiotic sensitivity pattern of the isolated A. veronii

The antibiotic susceptibility of the isolated *A. veronii* was checked according to the method of Bauer et al. [18]. The isolated *A. veronii* was cultured on brain heart infusion (Oxoid Ltd., UK) broth for overnight incubation at 30ºC. The MacFarland No. 0.5 standard solution was used for comparing the turbidity of the fresh culture through continuous dilution. Then, the fresh culture of desired turbidity was spread onto triplicate Mueller-Hinton agar media (Oxoid Ltd., UK). Antimicrobial agents, *viz*., tetracycline (30 µg/disk), oxytetracycline (30 µg/disk), chlortetracycline (25 µg/disk), ciprofloxacin (5 µg/disk), streptomycin (10 µg/disk), gentamicin (10 µg/disk), neomycin (30 µg/disk), erythromycin (15 µg/disc), and ampicillin (20 µg/disc) (Oxoid Ltd., UK) were placed onto the bacterial culture and incubation at 37ºC for 24–48 h. Finally, the inhibition zone diameter was determined in mm.

### Determination of the pathogenicity of the isolated A. veronii in healthy stinging catfish by aquarium-based experimentally induced infection

Forty apparently healthy adult stinging catfish (average body weight: 60 gm) were collected from a commercial pond. The fish were housed individually in a glass tank with a capacity of 120 l at the Bangladesh Fisheries Research Institute, Mymensingh. They were maintained in this habitat for 3 weeks, with an initial week dedicated to allowing the fish to acclimatize to their surroundings. The fish were subjected to a 24-h period of food deprivation before the introduction of the pathogen. The water was continuously monitored and refreshed daily, with a 50% replacement rate of fresh water. The YSI 85 instrument was utilized to test various water quality parameters, including temperature (29ºC ± 1.2ºC), dissolved oxygen (5.8 ± 1.2 mg/l), pH (7.2 ± 0.3), and ammonia concentration (0.3 ± 0.1 mg/l) [19]. The experimental conditions remained constant throughout the study. The initial isolate of *A. veronii*, obtained from the liver of stinging catfish, was utilized in the experimental infection. The pure isolate was subjected to culturing [19], and then the suspensions were diluted to obtain a concentration of 1 × 10^7^ CFU/ml through a 10-fold serial dilution using 0.85% saline solution [20]. Three experimental trials were devised, referred to as the first, second, and third groups of stinging catfish. Each experimental trial group was, thereafter, subdivided into three groups: oral, intraperitoneal (IP), and fake control. Each group consisted of five fish housed in a 120-l glass aquarium. The oral and IP groups were administered with a 1 ml inoculum containing 1 × 10^7^ CFU/ml of *A. veronii*, while the remaining group received an injection of 1 ml phosphate buffered saline (PBS) as a sham control. The fish were subjected to daily observation over 14 days following infection to detect any clinical indications, anomalous behavior, or instances of mortality.

### Accession numbers

The GenBank submission of the analyzed data on the full genome sequence of the *A. veronii* strain (strain Alim_AV_1000), isolated from Shing fish in Bangladesh, was accomplished under the Bioproject accession numbers PRJNA810265, Biosample accession number: SUB11126221, and accession number: JALLKR000000000.

## Results 

### Bacterial identification and genome analysis

Based on MALDI-TOF-MS, strain Alim_AV_1000 was identified as *A. veronii*. The comprehensive genome analysis service (CGAS) was explored to interpret the assembled genome. The CGAS genome analysis revealed that the genome’s average G+C content was 58.87%, with 93 contigs totaling 4,494,515 bp (Table S1). The Alim_AV_1000 strain was determined to be ST 492 using multilocus sequence typing.

The rapid annotation using subsystem technology tool kit was used to annotate *A. veronii* Alim_AV_1000. A total of 4,229 protein-coding sequences (CDS), 102 transfer RNA (tRNA) genes, and 13 ribosomal RNA (rRNA) genes have been found in this genome. The annotated characteristics have been represented in Table S2. The annotation revealed a total of 3,365 proteins with functional assignments and 864 proteins that were hypothesized (Table 1).

**Figure S1.**
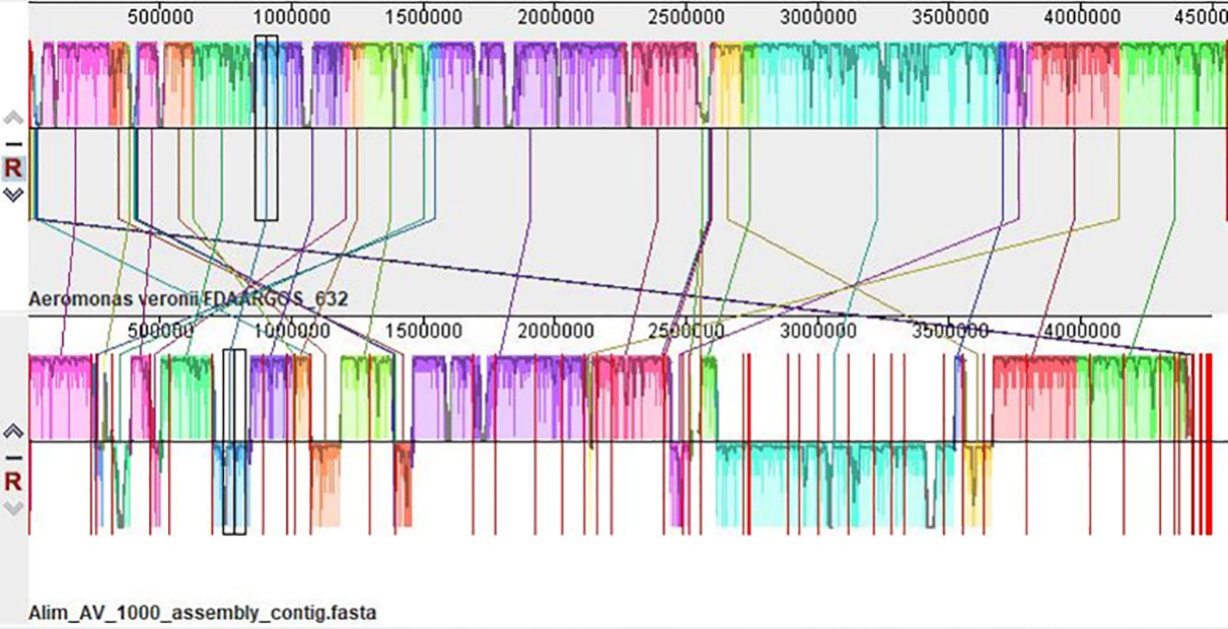
Synteny analysis between Alim_AV_1000 and *A. veronii* reference strain. Blocks with similar color represent similar sequence.

**Figure 1.**
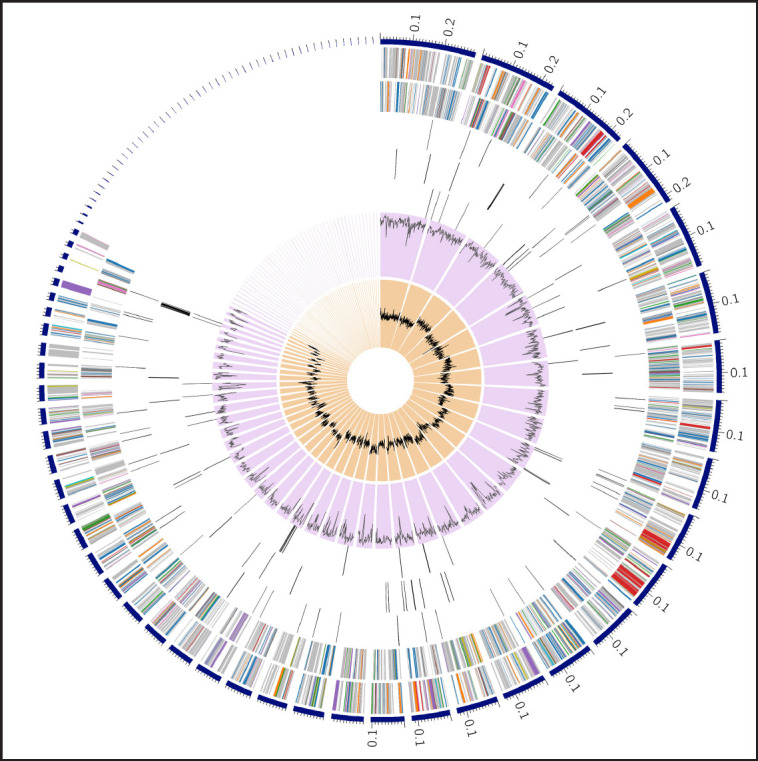
Distribution of genome annotation of *A. veronii* strain Alim_AV_1000 using circular graphical display. Listed in order from periphery to central rings, indicating contigs, forward strand CDS, reverse strand CDS, RNA genes, homologous CDS for approved genes for antibiotic resistance, homologous CDS for approved virulence associated factors, G-C content, and G-C skew.

**Figure 2.**
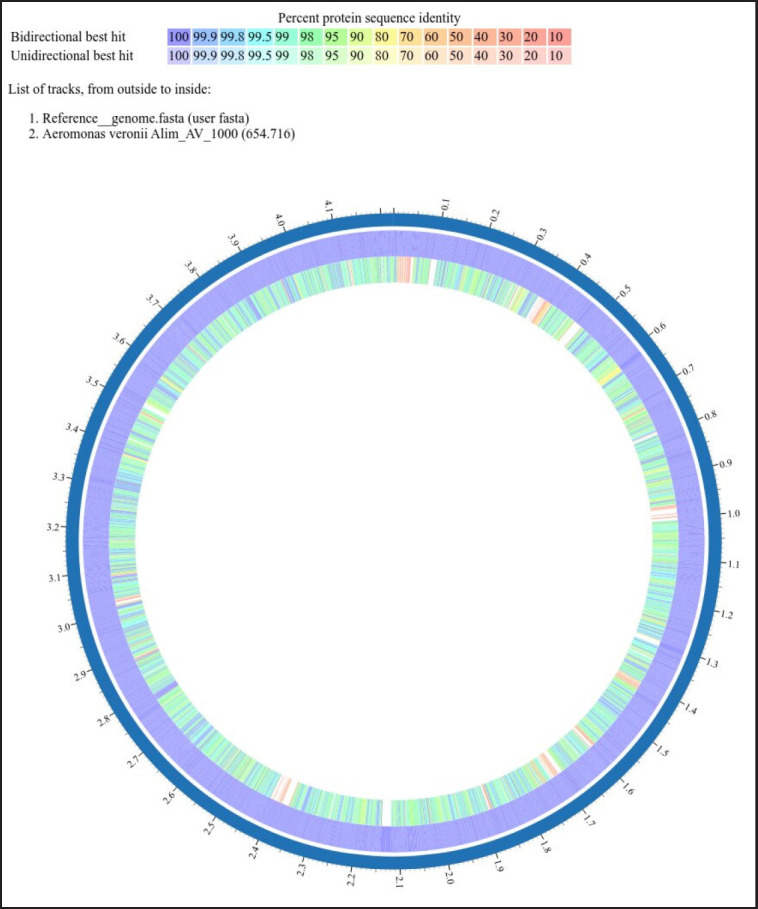
Proteome analysis of *A. veronii* strain Alim_AV_1000; ≥95% are conserved between this isolate and reference strain.

The synteny analyses between this isolate and the reference genome of *A. veronii* showed that the genome structure of Alim_AV_1000 resembles the reference genome (Fig. S1). The similar-colored boxes reflect the same genetic structure, while the white spaces represent areas unique to each individual isolate. The crossed lines represent the orientation of a similar genetic structure. Among the proteins that had been assigned functions, 1,015 had been given Enzyme Commission numbers, 839 had been assigned to gene ontology (GO), and 730 had been mapped to Kyoto Encyclopedia of Genes and Genomes pathways. According to the annotation, 4,063 PLFams are unique to a single genus (genus-specific protein families) and 3,996 PGFams that span multiple genera (cross-genus protein families) in this genome. The scatterplot of genomic annotations is shown graphically in a sphere in Figure 1. The peripheral to central rings list contigs, forward and reverse CDS, RNA genes, CDS with homology to approved antibiotic resistance genes and approved virulence-associated factors, G-C content, and G-C deviation. The subsystem to which a certain set of genes belongs is indicated by the color of their coding region on both strands (forward and reverse). The projected proteome of the strain was compared to that of a reference strain (NZ_CP044060.1), and the whole genome sequence of *A. veronii* was determined. Among the anticipated gene products encoded on the reference chromosome, ≥95% are conserved between this isolate and the reference strain (Figure 2).

**Table S1.** Assembly details.

Parameters	Quantity
Contigs	93
GC content	58.87%
Plasmids	0
Contig L50	12
Genome length	4,494,515 bp
Contig N50	150,337
Chromosomes	0

**Table S2.** Annotated genome features.

Parameters	Quantity
CDS	4,229
tRNA	102
rRNA	13
Partial CDS	0
Miscellaneous RNA	0
Repeat regions	0

**Table 1.** Protein features.

Parameters	Quantity
Hypothetical proteins	864
Proteins with functional assignments	3,365
Proteins with EC number assignments	1,015
Proteins with GO assignments	839
Proteins with pathway assignments	730
Proteins with PATRIC genus-specific family (PLfam) assignments	3,996
Proteins with PATRIC cross-genus family (PGfam) assignments	4,063

### Subsystem analysis

**Figure 3.**
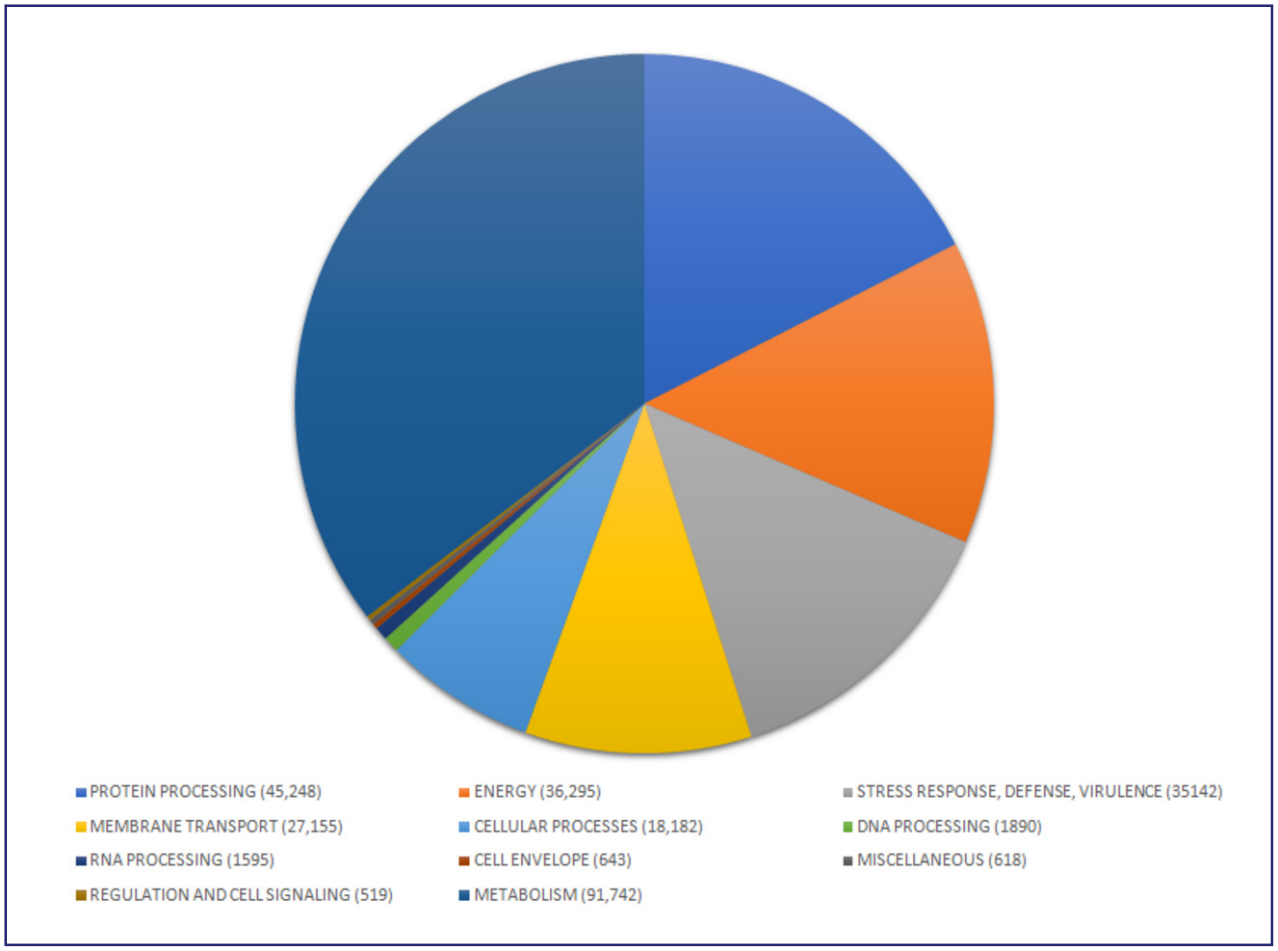
An overview of the subsystems for *A. veronii* strain Alim_AV_1000 genome.

A group of proteins that work together to carry out a specific biological activity or create a structural complex is called a subsystem. Figure 3 depicts a comprehensive overview of the various biological subsystems that are inherent within this particular genome.

### Specialty gene 

Several genes, annotated in the dataset, have been identified as homologs, exhibiting characteristics such as being proven transporters, factors responsible for virulence, pharmacological targets, and antimicrobial resistance (AMR) genes. Table 2 presents the homology data, comprising various genes and the specific database from which it was obtained.

### Antibiotic sensitivity and resistome analysis

The *A. veronii* isolate showed resistance patterns to all antimicrobial agents except two used in the study (Table 3).

The sequence of several antibiotic-resistant genes (ampicillin, tetracycline, and ciprofloxacin) in the strain was determined, which was an indication of multidrug resistance. The genes linked to AMR, annotated in this genome, and the accompanying mechanisms of AMR are presented in Table 4.

### Phylogenetic analysis

**Figure 4.**
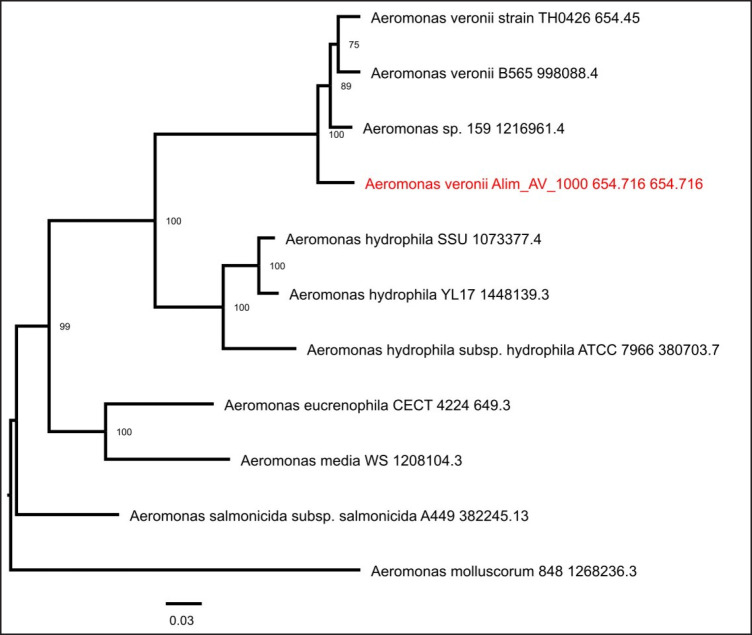
Whole genome phylogeny analysis of *A. veronii* strain Alim_AV_1000.

Phylogenetic analysis of *A. veronii *Alim_AV_1000 shows that it is highly similar to isolates TH0426 and strain B56 isolated from catfish in China (Figure 4).

### Pathogenicity and virulence factors 

The onset of clinical manifestations, such as pinpoint hemorrhages, ulceration, and muscular erosion in the tail region, was observed in stinging catfish belonging to each of the three trial groups (first to third). These fish were experimentally infected with *A. veronii* either orally or via the IP route of infection on the first day postinfection (p.i.), and subsequently experienced mortality until the sixth day of p.i. The study observed the highest mortality rate (100%) among the catfish in all three trial groups (first to third trial groups) throughout the period from the first to sixth day, following infection via the IP (i.p.) route (Table 5). The mortality rate observed among catfish in the first to third trial groups, when infected by the oral route, ranged from 80% to 100% (Table 5). In contrast, it was noteworthy that not a single fish from the fake (PBS) control groups experienced mortality throughout the study. Throughout the 14-day observation period, the infected fish did not exhibit any clinical indications of infection caused by *A. veronii*.

**Table 2.** Specialty genes.

Parameters	Sources	Genes
Antibiotic resistance	CARD	9
Antibiotic resistance	NDARO	2
Antibiotic resistance	PATRIC	38
Drug target	DrugBank	41
Drug target	TTD	7
Transporter	TCDB	34
Virulence factor	PATRIC_VF	17
Virulence factor	VFDB	7
Virulence factor	Victors	32

**Table 3.** Results of antibiotic sensitivity pattern of isolated *A. veronii*.

Name of antibiotic	Status of antibiotic
Tetracycline (30 µg/disc)	Resistant
Oxytetracycline (30 µg/disc)	Resistant
Chlortetracycline (25 µg/disc)	Resistant
Ciprofloxacin (5 µg/disc)	Resistant
Streptomycin (10 µg/disc)	Resistant
Gentamicin (10 µg/disc)	Susceptible
Neomycin (30 µg/disc)	Resistant
Erythromycin (15 µg/disc)	Susceptible
Ampicillin (20 µg/disc)	Resistant

**Table 4.** AMR genes.

AMR mechanism	Genes
Antibiotic activation enzyme	*Kat*G
Antibiotic inactivation enzyme	AAC (6´)-Ic, f, g, h, j, k, l, r-z, *Chp*A family
Antibiotic target in susceptible species	*Alr*, *Ddl*, *dxr*, EF-G, EF-Tu, *fol*A, *Dfr*, *fol*P, *gyr*A, *gyr*B, *Iso-tRNA*, *kas*A, *Mur*A, *rho*, *rpo*B, *rpo*C, *S10p*, *S12p*
Antibiotic target protection protein	*Qnr*B family
Antibiotic target replacement protein	*fab*V
Efflux pump conferring antibiotic resistance	*Mac*A, *Mac*B, *Mdt*L, *Tol*C/*Opm*H
Gene conferring resistance via absence	*gid*B
Protein altering cell wall charge conferring antibiotic resistance	*Gdp*D, *Pgs*A
Regulator modulating expression of antibiotic resistance genes	*H-NS*, *Oxy*R

The VFDB search results revealed that the most abundant virulence elements in the *A. veronii* strain of this study were adhesion genes, followed by secretion systems and toxins (data not shown). In *A. veronii *Alim_AV_1000, we found two intact phage elements and one incomplete phage region.

## Discussion

Currently, WGS is an important tool widely used for accurate microbial identification, comparing genomes, and genetic characterization. It facilitates in-depth, high-resolution microbial characterization that includes antibiotic resistance, molecular epidemiology, and virulence. In this study, *de novo* assembly of Alim_AV_1000 generated a whole genome consisting of 4,494,515 bp, which is a nearly complete genome. The 41 publicly available *A. veronii *strains contain 8,710 genes in the pan-genome and 2,855 genes in the core genome. Moreover, 58.1%–58.9% average G+C content and 4.28–4.95 Mb full genome size [12].

**Table 5.** Determination of pathogenicity of the isolated *A. veronii* in the healthy stinging cat fishes by aquarium-based experimentally induced infection.

Experimental groups	Hosts	Fish No.	Strain	Routes and doses			Number and % mortality of fishes (Day 1-day 6 of p.i.)			Number and % of recovered fishes (Day 7-day 14)		
				Oral (0.1 ml) (107 CFU/ml)	IP (0.1 ml) (107 CFU/ml)	Mock control group IP injection PBS (0.2 ml/dose)	Oral	IP	Control	Oral	IP	Mock control group (PBS)
First trial	Healthy cat fish	15	*Aeromonas veronii* (field isolate)	5	5	5	4(80%)	5(100%)	0(0%)	1 (20%)	0 (0%)	0(0%)
Second trial		15		5	5	5	5(100%)	5(100%)	0(0%)	0 (0%)	0 (0%)	0(0%)
Third trial		15		5	5	5	4(80%)	5(100%)	0(0%)	1 (20%)	0 (0%)	0(0%)

AMR represents a pervasive global challenge that encompasses several dimensions of health, spans multiple industries, and exerts a profound influence on society as a whole. In this study, the PATRIC Annotation Service was explored to detect AMR genes in this genome, which is a k-mer-based detection method. In this case, PATRIC’s collection of curated, integrated, and mapped antibiotic resistance data (representative AMR gene sequence variants) [16]. PATRIC’s database for AMR genes assigns each AMR functional role, broad mechanisms underlying AMR, antimicrobial classes, and even specific antimicrobials that impart resistance in some circumstances. The phenotypic manifestation of AMR genes is not necessarily indicated by the existence, even in full size, of AMR-associated genes in a particular genome. Specific AMR mechanisms and the presence or lack of SNP mutations that represent resistance should be considered while evaluating AMR pathways. Both phenotypic and genotypic data revealed that the Alim_AV_1000 strain is a multidrug-resistant strain. Similar to our study, multidrug-resistant *A. veronii* was isolated in India and showed resistance to several antibiotics such as ampicillin, ciprofloxacin, colistin, and trimethoprim [21]. Moreover, river water isolates of *A. veronii* in Italy also showed resistance to various antimicrobial agents, such as levofloxacin, colistin, and pipercillin [22]. The *A. veronii* strains are resistant to several antimicrobial agents, reflecting the significant role of the environment in disseminating the resistant strains.

As an integral component of the comprehensive genome analysis report, incorporation of both reference and representative genomes was made for the phylogenetic analysis, encompassing them within the scope of the phylogenetic investigation. The Mash/MinHash algorithm was employed to ascertain the most similar reference and representative genomes [23]. Genome-wide global protein families (PGFams) were used to define this genome’s phylogenetic location [24]. The protein sequences were aligned based on a comparison of similar families using the MUSCLE software [25], and the nucleotides were subsequently mapped onto the resulting protein alignment. Data matrices were made by concentrating the joint set of amino acid and nucleotide alignments, and RaxML [26] was utilized to evaluate this matrix to generate support values in the tree. Fast bootstrapping [26] was used. Phylogenetic analysis showed that the *A. veronii* genome in this study might have originated from China catfish isolate TH0426 or strain B56 (Figure 4). This is probably due to the huge fish hatchery trade between these two countries.

It is well recognized that the pathogenicity of *A. veronii* is complex and multifactorial, and several virulence-associated factors play a role in the clinical manifestations. Virulence factors include the adhesion system, biologically active substances, the presence of a flagella, the secretion system, surface polysaccharides, iron-binding mechanisms, and extracellular factors such as enzymes and toxins [27]. Adhesions play a role in initiating infection by colonizing the host, which is considered the most important step in the pathobiogenesis of a bacterium [28]. *Aeromonas veronii *Alim_AV_1000 encodes several flagella, pili, and fimbriae genes, contributing to adherence, motility, and biofilm formation in the pathobiogenic process [28]. A type III secretion system (TSS) was also detected and is widespread [29]. Alim_AV_1000 contains several TSS secretion systems (T2SS, T3SS, and T6SS). Among different TSS systems, T2SS is well recognized for secreting several proteins that contribute to virulence [29]. The T3SS system is responsible for the secretion and translocation of toxins and effector proteins into the host cell [29]. T3SS is recognized as the pathogenic phenotype of *Aeromonas*, a major virulence attribute of *A. salmonicida *[30]. Another member of TSS is T6SS, which serves as a virulence-associated factor and is responsible for strengthening the bacterium’s capability while interacting with the environment, host, and other competitor bacteria [30]. It is reported that most of the *A. veronii* genome encodes the T6SS system [12]. This isolate has another virulence gene, *Aeromonas *secretin (*Asc*) (encoded by *Asc*, or Asc; comprised of AscD, AscJ, AscV, AscF, AscR, AscS, AscT, and AscU), that plays a crucial and important role in bacterial T3SS [30]. This strain also encoded the Aop (*Aeromonas *outer protein) gene, released by the bacteria and injected into the host cell [30].

The virulent strains of *Aeromonas* spp. play a significant role as a causative agent in the outbreaks of MAS in the aquaculture industry in Bangladesh. Experimental infection by injection of *A. veronii* strain Alim_AV_1000 at 10^7^ CFU/ml manifested clinical signs and caused mortality. Similar to our study, *A. veronii* strain ML09-123 showed mortality by the experimental routes of infection in catfish [12].

Bacteriophages have a role in bacterial evolution and horizontal gene transfer in various ways, including the transfer of virulence genes. Moreover, prophage regions have been shown to play a role in the adhesion, invasion, and survival strategies of bacteria through specific mechanisms. Similar to our findings, catfish isolates TH0426 from China and ML09-123 from the United States had different counts of bacteriophage elements but shared whole phage components [12].

## Conclusion

The WGS data of this study provided a comprehensive understanding of the genomic characterization of an *A. veronii* isolates. Moreover, the genomic data from the *A. veronii* Alim_AV_1000 strain will help researchers unravel the molecular mechanisms behind the spread of antibiotic resistance within the Aeromonadaceae family. Phylogenetic analysis revealed the close relationship of this strain with China strain TH0426 and strain B65. Future research could be performed on the isolation, molecular characterization, and comparative genomics of more *A. veronii* strains from aquatic animals from different regions of Bangladesh.

## List of Abbreviations

AMR, Antimicrobial resistance; *Asc*, Aeromonas secretin; MALDI-TOF MS, Matrix-assisted laser desorption/ionization time-of-flight mass spectrometry; MAS, motile *Aeromonas *septicemia; PHASTER, Phage search tool enhanced release; VFDB, virulence factor database; WGS, Whole genome sequencing.
